# Connectome-Based Biomarkers Predict Subclinical Depression and Identify Abnormal Brain Connections With the Lateral Habenula and Thalamus

**DOI:** 10.3389/fpsyt.2019.00371

**Published:** 2019-06-12

**Authors:** Yunkai Zhu, Shouliang Qi, Bo Zhang, Dianning He, Yueyang Teng, Jiani Hu, Xinhua Wei

**Affiliations:** ^1^Sino-Dutch Biomedical and Information Engineering School, Northeastern University, Shenyang, China; ^2^Key Laboratory of Medical Image Computing of Northeastern University (Ministry of Education), Shenyang, China; ^3^Department of Radiology, Wayne State University, Detroit, United States; ^4^Department of Radiology, Guangzhou First People’s Hospital, School of Medicine, South China University of Technology, Guangzhou, China

**Keywords:** resting state functional MRI, brain network, subclinical depression, brain biomarker, functional connection, node degree

## Abstract

Subclinical depression (SD) has been considered as the precursor to major depressive disorder. Accurate prediction of SD and identification of its etiological origin are urgent. Bursts within the lateral habenula (LHb) drive depression in rats, but whether dysfunctional LHb is associated with SD in human is unknown. Here we develop connectome-based biomarkers which predict SD and identify dysfunctional brain regions and connections. T1 weighted images and resting-state functional MRI (fMRI) data were collected from 34 subjects with SD and 40 healthy controls (HCs). After the brain is parcellated into 48 brain regions (246 subregions) using the human Brainnetome Atlas, the functional network of each participant is constructed by calculating the correlation coefficient for the time series of fMRI signals of each pair of subregions. Initial candidates of abnormal connections are identified by the two-sample *t*-test and input into Support Vector Machine models as features. A total of 24 anatomical-region-based models, 231 sliding-window-based models, and 100 random-selection-based models are built. The performance of these models is estimated through leave-one-out cross-validation and evaluated by measures of accuracy, sensitivity, confusion matrix, receiver operating characteristic curve, and the area under the curve (AUC). After confirming the region with the highest accuracy, subregions within the thalamus and connections associated with subregions of LHb are compared. It is found that five prediction models using connections of the thalamus, posterior superior temporal sulcus, cingulate gyrus, superior parietal lobule, and superior frontal gyrus achieve an accuracy >0.9 and an AUC >0.93. Among 90 abnormal connections associated with the thalamus, the subregion of the right posterior parietal thalamus where LHb is located has the most connections (*n* = 18), the left subregion only has 3 connections. In SD group, 10 subregions in the thalamus have significantly different node degrees with those in the HC group, while 8 subregions have lower degrees ( *p* < 0.01), including the one with LHb. These results implicate abnormal brain connections associated with the thalamus and LHb to be associated with SD. Integration of these connections by machine learning can provide connectome-based biomarkers to accurately diagnose SD.

## Introduction

On the depression severity continuum, subclinical depression (SD) is a mild condition considered to be the precursor to major depressive disorder (MDD) (1, 2). Subjects with SD are very vulnerable to depression and are apt to generate suicide ideation (3, 4). The increasingly high incidence of SD among both college students and the elderly (estimated as high as 15%) clearly demonstrates the need for intensive investigation (5–7). Unfortunately, knowledge of neural substrates of SD is incomplete, making it difficult to identify reliable diagnostic biomarkers and take preventative treatments (8).

Some dysfunctional brain regions and connections have been evaluated in order to identify new biomarkers for SD. *Via* resting state fMRI (rs-fMRI), we have previously demonstrated that the altered spontaneous neuronal activity by measurement of amplitude of low-frequency fluctuations (ALFF) and disrupted functional connectivity (FC) are implicated in SD (9–11). We also found that SD presents the increased interhemispheric FC and cortical degree centrality, as well as decreased subcortical degree centrality. These measures differentiate SD subjects from healthy controls (HCs) (10–12). SD is characterized by changed FCs between subregions of the anterior cingulate cortex (ACC), increased FC of Hb within default model network regions, and decreased FC within salience network regions (8, 13). Kaiser et al. (14) demonstrated that there exists a high correlation between the neural activity of dorsal anterior cingulate cortex (dACC) and posterior cingulate cortex (PCC) in SD subjects, indicating that SD subjects are confronted with greater difficulty of shifting out of internally directed and ruminative thinking. Dedovic et al. (15) and Petrican et al. (16) reported the weaker functional dominance in dorsal attention network (DAN) [low connectivity between the superior parietal lobule (SPL) and the frontoparietal control network].

Network neuroscience explores interactions of different neurobiological element from an integrative perspective and is capable of providing with better predictive biomarkers for brain disorders by machine learning (17, 18). Machine learning is suitable for individual-level prediction from a prospective viewpoint, and it is a potentially powerful tool for precision psychiatry (19). For example, Support Vector Machine (SVM), as a typical method of machine learning, has been widely used to identify imaging biomarkers in diseases such as schizophrenia, major depression, bipolar disorder, etc. (20). For more information on machine learning and its application in psychiatry, one can refer to the comprehensive reviews (21–24). Recently, machine learning has proved useful to build connectome-based biomarkers for autism spectrum disorder, bipolar disorder, subtypes of depression, and schizophrenia (25–28). However, not many connectome-based biomarkers have been developed for SD.

Compared with SD, MDD has received more attention and significant breakthroughs have been achieved. For example, concrete evidence has demonstrated that bursts within the lateral habenula (LHb) drive depression in rats (29). As an evolutionary conserved epithalamic structure, LHb is involved in negative motivational value and decision-making (30–33). LHb is also considered to be the pathophysiological basement of MDD (34, 35). For more details on LHb, one can refer to these recent reviews (36–38). The deep brain stimulation of LHb has been successfully used to treat patients with refractory MDD (39). These findings on MDD may provide useful clues regarding SD.

LHb has been investigated by multimodal MRI in depressive and healthy subjects, but not in subjects with SD. LHb volume measured by high-resolution T1-weighted images decreases in depression, but not in posttraumatic stress disorder or schizophrenia (40, 41). Using task-based functional MRI (fMRI), Salas et al. (42) have shown that LHb is activated in response to negative reward prediction. It is worth noting that the fMRI study on LHb has several limitations. First, the habenula volume approximately ranges from 29 to 36 mm^3^ in each hemisphere based on structural MRI and postmortem measurement, which can be smaller than the voxel size of standard fMRI (40, 41, 43). The smoothing kernels [5–12 mm full width at half maximum (FWHM)] are larger in size than LHb. Second, the habenular signal is likely contaminated by adjacent structures, such as the medial dorsal thalamus or the epithalamic paraventricular nucleus (43).

Herein, connectome-based biomarkers are developed to predict subclinical depression through a machine learning algorithm and identify dysfunctional brain regions and connections. The method of predictive modeling used in our study is different with the traditional method of brain mapping (13). Predictive modeling integrates all brain data or features into a single prediction of outcome, making multiple comparisons unnecessary and increasing statistical power (18). Specifically, we parcellate the whole brain into 48 regions and 246 subregions using the latest human Brainnetome Atlas (44) and build large-scale resting-state functional brain networks using fMRI data. A two-sample *t*-test is used to identify initial candidate connections, and the resultant regional connections are input into SVM models as features. The performance of the predictive models is estimated by leave-one-out cross-validation. The node degree of subregions within the thalamus is compared between SD and HC groups. Connections linked with subregions of LHb are further investigated.

## Materials and Methods

### Participants

All the participants were enlisted from volunteers who had undergone health screening at Guangzhou Medical University from 2012 to 2014. The Beck Depression Inventory II (BDI-II) scale is administered to evaluate the depression symptom severity. Thirty-four subjects (11 males, 23 females) with BDI-II score >13 are placed into the SD group (BDI score mean ± SD: 22.58 ± 6.92) and 40 healthy controls (21 males, 19 females) are selected to match the SD group by age, sex, and education. According to the two-sample *t*-test, there is no significant difference for the age (years) between SD and HC groups (mean ± SD: 19.91 ± 1.64 vs. 19.70 ± 0.85, *p* = 0.50), neither for the education (years) (mean ± SD: 13.18 ± 0.58 vs. 13.08 ± 0.62, *p* = 0.47). By the chi-square test, there is no significant difference for the gender (*p* = 0.07). None of the participants fulfilled the criteria for MDD based on *Diagnostic and Statistical Manual of Mental Disorders IV* (*DSM-IV*). Other inclusion criteria for all participants include age ranging from 19 to 25 years, right-handedness, no visualized lesion on any MRI scans, no neurological illness, and no alcohol or drug dependence. The study is approved by the Medical Ethics Committee of Guangzhou First People’s Hospital of Guangzhou Medical University and is in accordance with the 1964 Helsinki declaration and its later amendments or comparable ethical standards. All participants signed a written informed consent in accordance with the Declaration of Helsinki (2000).

### MRI Imaging Data Acquisition

All MRI images were acquired using one 3-Tesla MRI scanner (Siemens, Erlangen, Germany) with an eight-channel phase-array brain coil. Foam pads and headphone were utilized to minimize the head motion and reduce noise, respectively. As in our previous studies (10–12), high-resolution T1-weighted images were obtained with a standard magnetization prepared rapid gradient echo (MP-RAGE) sequence [repetition time (TR)/echo time (TE) 2,530/2.34 ms, flip angle (FA) 7°, field of view (FOV) 256 × 224 mm, slice thickness 1.0 mm]. The resting-state fMRI data were collected by one echo-planar imaging (EPI) sequence (TR/TE 2,500/21 ms, FA 90°, FOV 200 × 200 mm, matrix 64 × 64, 42 slices without gap, voxel size 3.5 × 3.1 × 3.1 mm). The images of 200 time points were collected, and the total amount of fMRI acquisition time is 500 s. During the resting-state fMRI scan, the participants were asked to relax, to close their eyes, not to think of anything in particular, and not to fall asleep. Wakefulness of participants has been confirmed immediately after the fMRI scanning session.

### Study Design and Main Procedures

**Figure 1:**
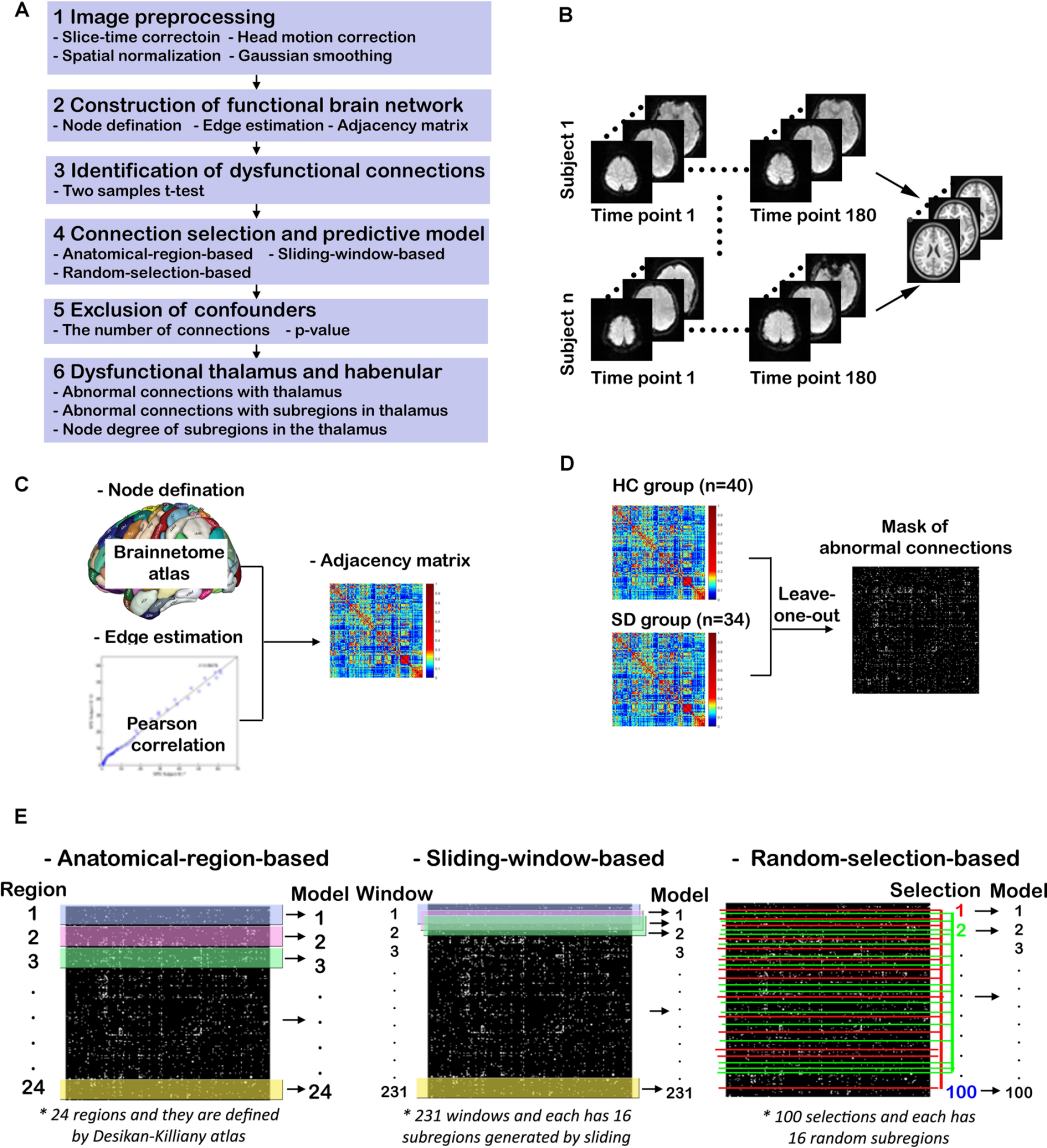
Study design and procedures. **(A)** Overview of the study procedures; **(B)** functional MRI (fMRI) image preprocessing; **(C)** construction of functional brain networks; **(D)** identification of dysfunctional connections; **(E)** connection selection and predictive models.

The study design and procedures are schematically shown in **Figure 1**. There are six steps for this study (**Figure1A**). After the first step of image preprocessing, functional brain networks for HCs and SDs are constructed. Then two-sample *t*-tests are used to identify potential dysfunctional connections. Three methods are proposed to further select connections from previously identified candidates. These selected connections are used to train and test the predictive models of SD. After excluding confounders such as the number of connections and *p*-value, dysfunctional brain regions and connections are determined by examining the models with high predictive accuracy. Finally the emphasis is placed on the dysfunctional thalamus and LHb. Abnormal connections associated with the thalamus and its subregions, including LHb, as well as the node degree of these subregions are characterized. These six steps are described in details below.

#### Functional MRI Image Preprocessing

As shown in **Figure 1B**, the T1-weighted and rs-fMRI data is preprocessed using the DPARSF toolbox (http://www.restfmri.net/forum/DPARSF) (45, 46) as follows. First, the initial 20 time points of raw fMRI data are removed in order to eliminate unstable factors. Second, the time layer correction and head movement correction are carried out. Third, the brain of each subject is registered to a normative template through spatial standardization. Fourth, a band-pass filtering of 0.01–0.1 Hz and Gaussian smoothing with 6 mm FWHM are implemented.

#### Construction of Functional Brain Networks

The procedure for constructing functional brain networks is shown in **Figure 1C**. First, the newly developed human Brainnetome Atlas is used to parcellate the whole brain into 48 brain regions (246 subregions). This atlas is four to five times as accurate as the traditional Brodmann map and has a more objective and accurate boundary (44). Each subregion represents a node in the constructed brain network. The time course of each subregion is calculated by averaging the time course of all voxels therein. The strength of functional connection or the connection weight (*W* *_ij_*), also identified as edge weight, is expressed as the Pearson correlation coefficient between the time courses of any two subregions (*i*, *j*). The correlation matrix is transformed into *Z* scores by applying Fisher’s *r*-to-*Z* transformation. For each individual, a weighted undirected network is obtained in the form of a 246 × 246 adjacency matrix (*A*). Given that it is controversial for interpreting negative correlation or functional connectivity (47, 48), the normalized absolute value of the matrix is used as done in previous studies (49, 50), such that 0 ≤ *W* *_ij_* ≤ 1 for all *i* and *j*.

Node degree and edge weight are used to determine whether a brain region (or subregion) is connected or dysfunctional in SD. The node degree (*k* *_i_*) refers to the number of connections that link this node to the rest of the network. For our weighted networks, the definition can be transformed as

(1) $$k_{i}=\sum_{j\in N}W_{ij}$$

where *W* *_ij_* is the strength of the connection between node *i* and node *j*, and *N* is the set of nodes in the network. The edge weight (*W* *_ij_*) is an important measure for evaluating the alteration in the strength of a connection in SD.

#### Identification of Dysfunctional Connections

As shown in **Figure 1D**, two-sample *t*-tests are performed to examine significant differences between edge weight in SD and HC groups (*p* < 0.05). For multiple comparisons, the false discovery rate (FDR) is controlled by the linear step-up procedure introduced by Benjamini and Hochberg (51). To avoid the information leakage, the two-sample *t*-test is carried out after leaving one out, not for all subjects. This step generated 74 different masks of abnormal connections. Based on each mask, the work in Connection Selection and Predictive Models is done. However, for the group study in Dysfunctional Thalamus and Lateral Habenula, the two-sample *t*-test is done for all subjects.

#### Connection Selection and Predictive Models

The study uses the library for support vector machines (LIBSVM) toolkit developed by Professor Lin of Taiwan University (https://www.csie.ntu.edu.tw/∼cjlin/libsvm/), which integrates many functions such as kernel selection, parameter adjustment, and prediction. For the training of SVM classification model, the radial basis kernel function (RBF) is used. This kernel function provides a good classification for samples with nonlinear relationship between labels and features as expressed below.

(2) $$K\left(x_{i},x_{j}\right)=\exp\left(-\gamma {\left|\left|x_{i}-x_{j}\right|\right|}^{2}\right),\gamma >0$$

According to the recommendation from LIBSVM, the values of the optimal penalty coefficient *C* and the kernel function parameter γ are determined by the way of “grid-search” using cross-validation (52). After going through all the pairs of (*C*, γ) with *C* = 2^−5^, 2^−3^,…, 2^15^ and γ = 2^−15^, 2^−13^,…, 2^3^, the pair leading to the best cross-validation accuracy is found.

Three methods are proposed to select connections from identified candidates, and these selected connections are used to train and test the predictive SVM models of SD, as shown in **Figure 1E**. For the first method, the significantly altered connections associated with each brain region defined by the human Briannetome Atlas are used as input features. A total of 24 SVM models are built to predict SD, and they are named as anatomical-region-based models. The performance of these models is estimated through leave-one-out cross-validation using measures of accuracy, sensitivity, confusion matrix, receiver operating characteristic (ROC) curve, and the area under the curve (AUC). These models are ranked by accuracy. The brain regions leading to the models with an accuracy >0.90 are considered to be dysfunctional.

To determine whether models using connections associated with subregions not belonging to one specific anatomically well-defined brain region and with subregions that are anatomically nonadjacent can achieve comparable performance to the anatomical-region-based models, two more independent experiments are conducted. First, the method of sliding window with 16 subregions is employed to generate different input features and models. The reason why the number of subregions was set as 16 is that the thalamus leading to the predictive model of the highest ACC owns 16 subregions. As shown in the middle column of **Figure 1E**, to slide the window row by row throughout the adjacency matrix (246 × 246) will generate 231 windows (246 – 16 + 1 = 231). The models using the connections within each individual window as input features are named as sliding-window-based models. Second, a model is constructed using the functional connections within 16 randomly selected subregions as input features. A total of 100 similar models are generated and identified as random-selection-based models. The accuracy values of these three categories of models are compared.

#### Exclusion of Confounders

To estimate whether the performances of the anatomical-region-based models are dependent on the number of connections associated with the brain region and the *p*-value of these connections, their correlation coefficients are assessed. The distribution of connections in the model with the highest accuracy is investigated to explore whether these models with good performance are independent.

#### Dysfunctional Thalamus and Lateral Habenula

In order to further identify dysfunctional subregions and connections, the connections of brain regions achieving the highest predictive accuracy (thalamus) are examined. The number and *p*-value of connections associated with each of the 16 subregions are identified. Finally, the node degree of each subregions is compared between SD and HC groups.

## Results

### Anatomical-Region-Based Models

**Figure 2:**
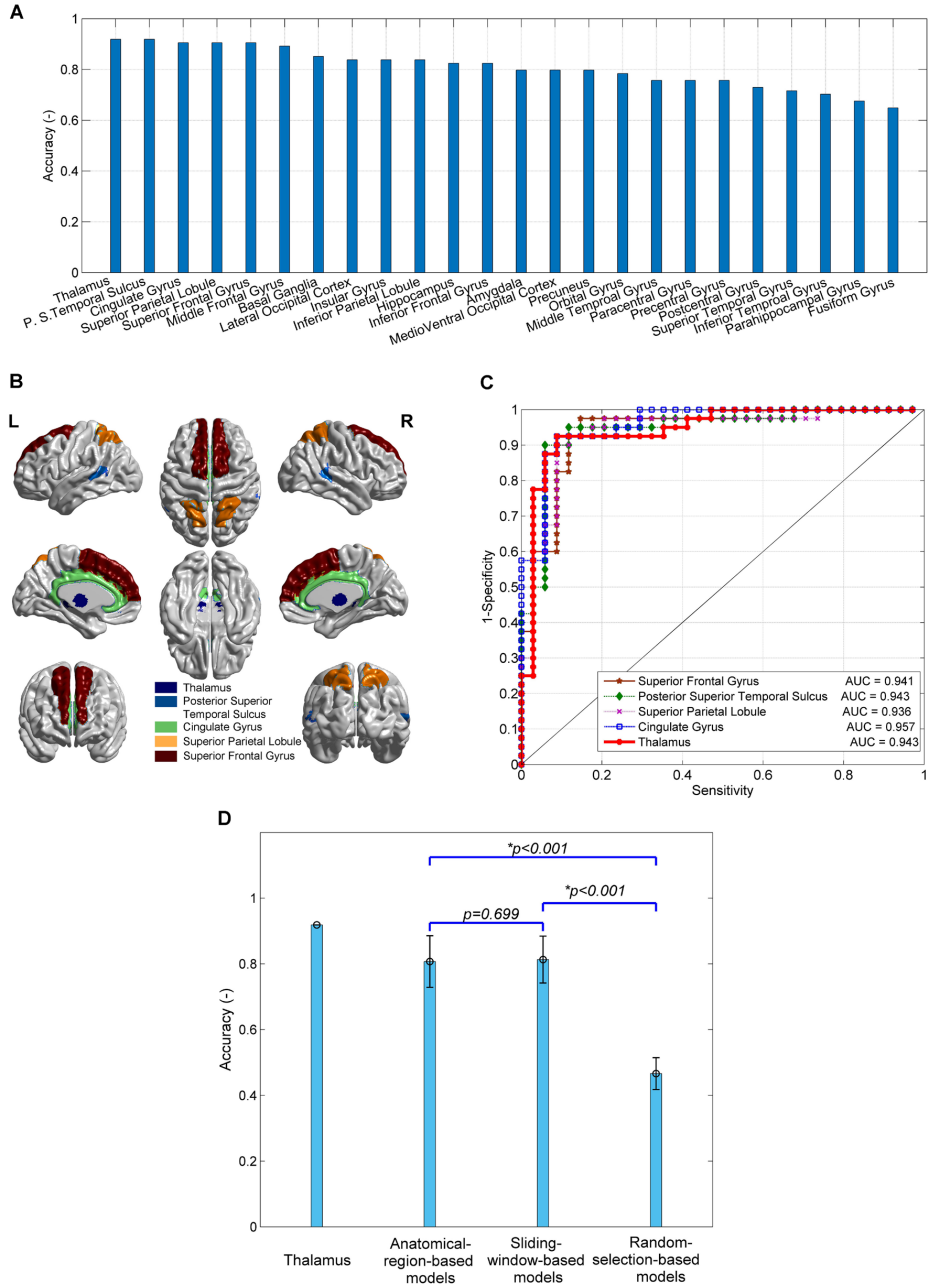
The performances of predictive models of subclinical depression (SD) and their comparison. **(A)** The prediction accuracy of 24 anatomical-region-based models; **(B)** the brain regions leading to the top five accuracy models; **(C)** receiver operating characteristic (ROC) curves and area under the curve (AUC) of the top five models; **(D)** comparison of the accuracy of models using connections with thalamus, 24 anatomical-region-based models, 231 sliding-window-based models, and 100 random-selection-based models.

The 24 anatomical-region-based models ranked by the accuracy of predicting SD are presented in **Figure 2A**. The accuracy ranges from 0.65 to 0.92. The top five models used connections associated with the regions of thalamus, posterior superior temporal sulcus, cingulate gyrus, superior parietal lobule, and superior frontal gyrus. The accuracy of each of these five models is higher than 0.90. The anatomical locations are shown in **Figure 2B**. The ROC curves and the AUC values are shown in **Figure 2C**. The cingulate model achieves the highest AUC of 0.957. The thalamus model yields the second highest AUC of 0.943. The confusion matrices of the top five anatomical-region-based models are listed in **Table 1**. For the thalamus model, 31 out of 34 subjects with SD (91.2%, also defined as sensitivity) and 37 out of 40 HCs (92.5%, also defined as specificity) are predicted accurately. The posterior superior temporal sulcus model yields the highest specificity of 95.0%, and the posterior superior temporal sulcus model yields the highest sensitivity.

**Table 1:** The confusion matrices of the top five anatomical-region-based models.

*Model* and items	Normal (gold standard)	Patient (gold standard)	Total
***Thalamus model***			
Predict to normal	37 (92.5%)	3 (8.8%)	40
Predict to patient	3 (7.5%)	31 (91.2%)	34
***Posterior superior temporal sulcus model***			
Predict to normal	36 (90.0%)	2 (5.9%)	38
Predict to patient	4 (10.0%)	**32 (94.1%)**	36
***Cingulate gyrus model***			
Predict to normal	37 (94.4%)	4 (11.8%)	41
Predict to patient	3 (5.6%)	30 (88.2%)	33
***Superior parietal lobule model***			
Predict to normal	36 (90.0%)	3 (11.1%)	39
Predict to patient	4 (10.0%)	31 (88.9%)	35
***Superior frontal gyrus model***			
Predict to normal	**38 (95.0%)**	5 (14.7%)	43
Predict to patient	2 (5.0%)	29 (85.3%)	31

### Other Subregion Selection Strategies

The accuracy of the models using other subregion selection strategies is compared with that of the anatomical-region-based models, as shown in **Figure 2D**. No significant difference in accuracy is observed between the 231 sliding-window-based models and the 24 anatomical-region-based models (0.81 ± 0.06 vs. 0.80 ± 0.08). The accuracy of the 100 random-selection-based models is 0.45 ± 0.06, which is significantly lower than that of the anatomical-region-based models and the sliding-window-based models (*p* < 0.001). The top five anatomical-region-based models, and in particular the thalamus model, achieve extraordinarily higher accuracy, as compared with the other models. Two important features are worthy to be noted. First, the brain regions involved in the top five anatomical-region-based models are potentially dysfunctional due to SD. Second, the arbitrary anatomically adjacent subregions (obtained by the sliding window method) can generate comparable prediction accuracy with anatomically well-defined subregions (obtained by the anatomical-region-based method), but the randomly selected subregions cannot reliably predict SD.

### Effect of the Number of Connections and the *p*-value

**Figure 3:**
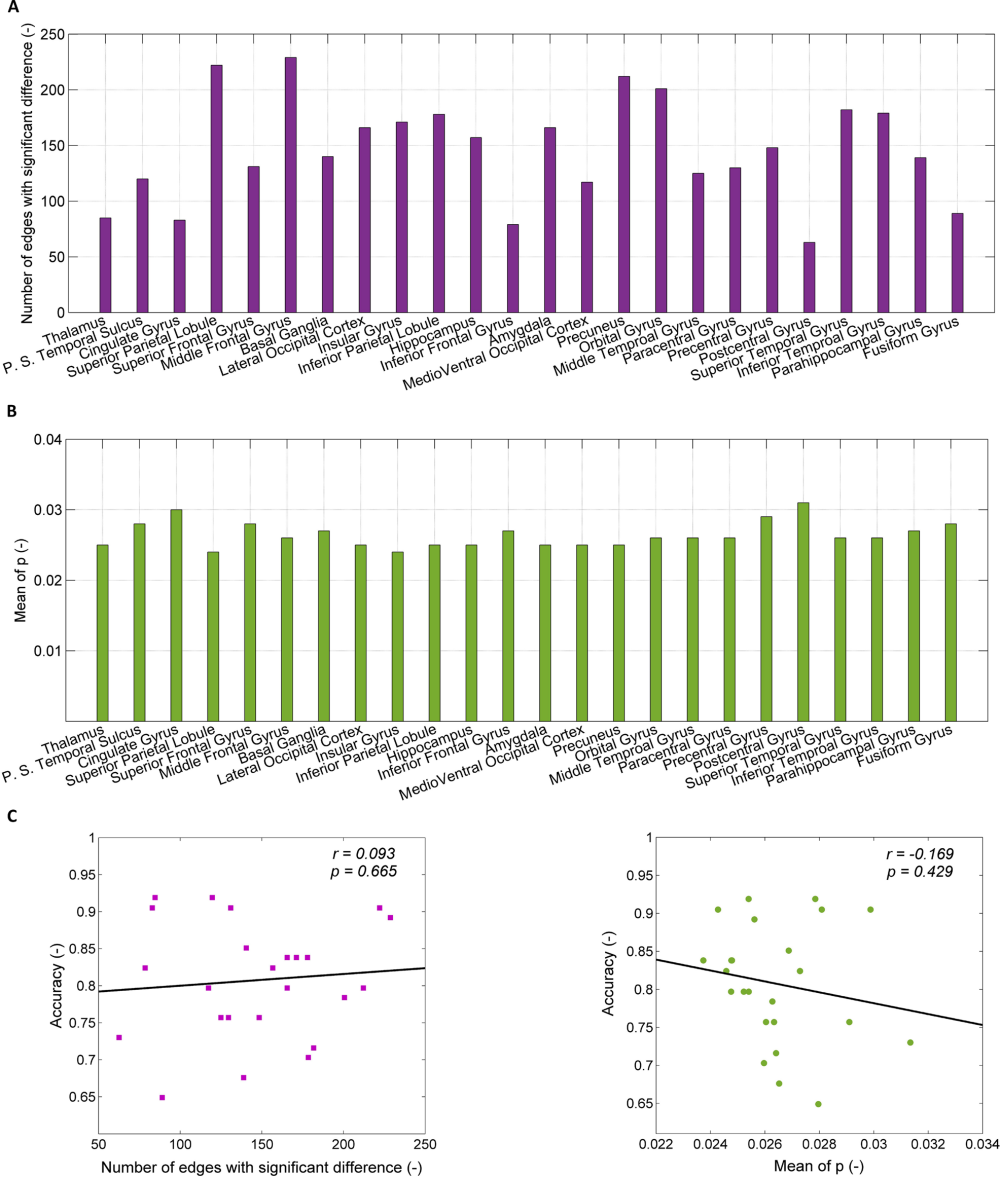
Exclusion of confounders (the number of connections and the mean *p*-value). **(A)** The number of connections with significant difference between healthy controls (HCs) and SDs for each of 24 brain regions; **(B)** the *p*-value for significant difference of connection weight between HCs and SDs for each of 24 regions; **(C)** the relationship between the accuracy of prediction and the number of connections with significant difference (left part), between the accuracy of prediction and the mean of *p*-value for significant difference of connection weight (right part).

The number of connections associated with the 24 brain regions used for the predictive models of SD ranges from 52 to 240, as shown in **Figure 3A**. The top five models, which correspond to the regions of thalamus, posterior superior temporal sulcus, cingulate gyrus, superior parietal lobule, and superior frontal gyrus, have connections with the average number of 85, 120, 83, 222, and 131, respectively. **Figure 3B** shows the mean *p*-value of connections in individual brain regions, which ranges from 0.025 to 0.031.

The dependence of the accuracy of the predictive models on the number of connections associated within individual brain regions and the mean *p*-value of connections are shown in **Figure 3C**. The accuracy of the predictive models is related to the number of connection associated with brain regions (*r* = 0.093), but there is no statistical significance (*p* = 0.665). When the number of connections associated with brain regions is randomly reduced to 90, as with the thalamus, the accuracy does not increase. For instance, after reducing the number of connections in middle frontal gyrus from 229 to 90, the accuracy decreases from 0.89 to 0.70 (the mean of 100 times random samples). The accuracy of the predictive models is negatively related to the mean *p*-value of connections associated with brain regions without statistical significance (*r* = 0.169, *p* = 0.429). The high value of accuracy of the top five models is due to neither the large number of connections, nor the small *p*-value of the connections.

### Dysfunctional Connections With the Thalamus

**Figure 4:**
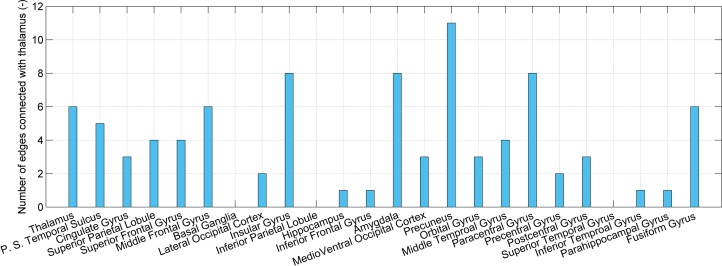
The number of connections that present significant difference and connect the thalamus and each of 24 brain regions.

Given that the thalamus is seen as one possible dysfunctional brain region of SD, connections associated with the thalamus are investigated, as shown in **Figure 4**. The number of connections between the thalamus and precuneus, insular gyrus, paracentral gyrus, and amygdala is higher than that of the other regions (11, 8, 8, and 8, respectively). However, the number of connections between the thalamus and itself, the posterior superior temporal sulcus, cingulate gyrus, superior parietal lobule, and superior frontal gyrus is only 6, 5, 3, 4, and 4, respectively. High accuracy values of the models using connections associated with the posterior superior temporal sulcus, cingulate gyrus, superior parietal lobule, and superior frontal gyrus are independent on the thalamus. These regions may also be impacted by SD.

### Subregions Within the Thalamus and Lateral Habenula

**Figure 5:**
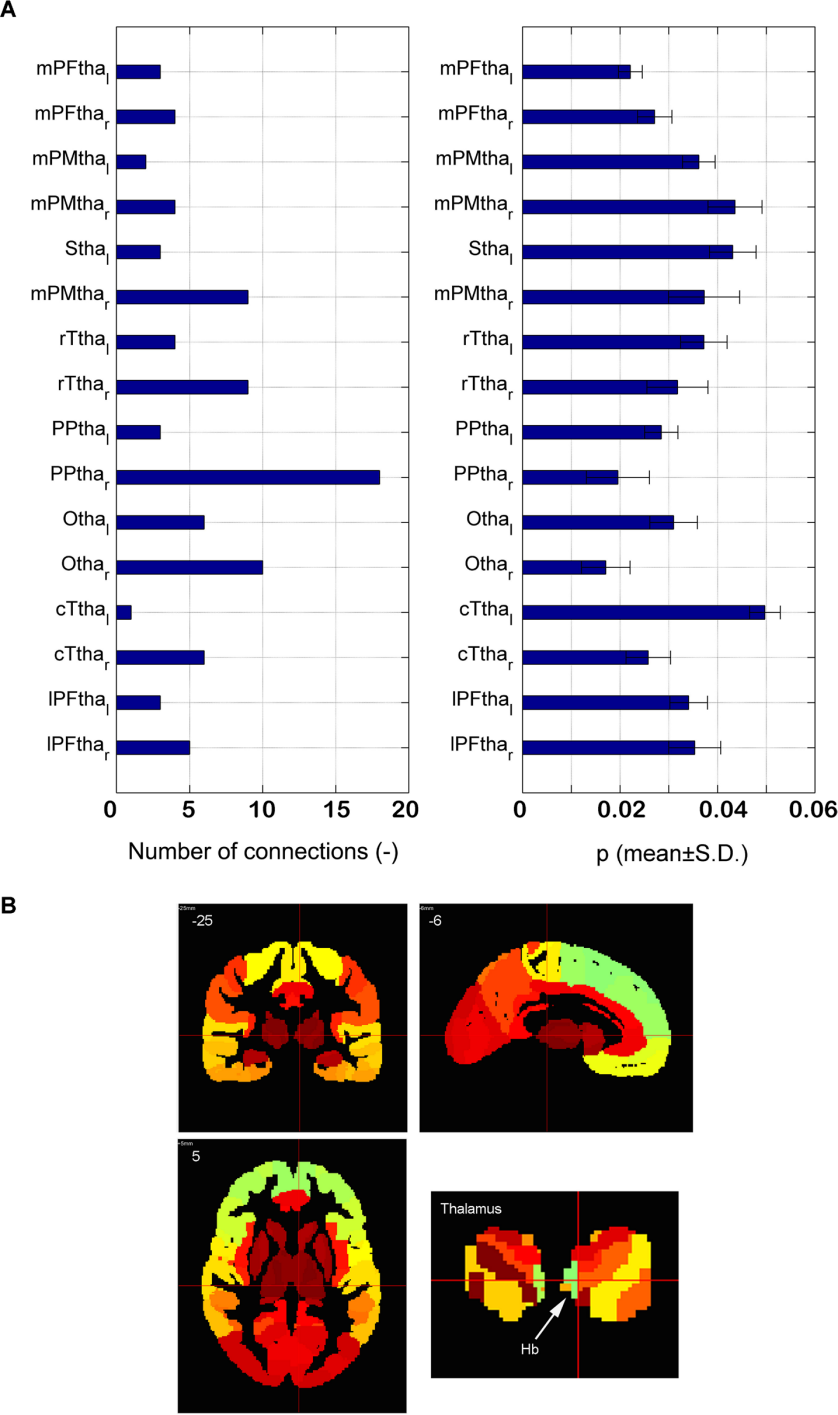
Dysfunctional thalamus and lateral habenula. **(A)** The number of dysfunctional connections for16 subregions of thalamus (the left part) and the *p*-value of the dysfunctional connections with 16 subregions of thalamus (the right part); **(B)** the anatomical atlas of thalamus and LHb.

The distribution of the 90 significantly different connections associated with the thalamus among 16 subregions is shown in **Figure 5A**. There are 18 connections associated with the right posterior parietal thalamus (PPtha_r), much higher than those connected with the other regions. The significant asymmetry is observed, i.e., the right side has more connections than the left. Astonishingly, only two edges are connected to the left posterior parietal thalamus. The *p*-value of connections associated with PPtha_r is smaller than that of PPtha_l, as illustrated in the right part of **Figure 5A**. Based on Montreal Neurological Institute (MNI) coordinates, LHb is located in the posterior parietal thalamus (**Figure 5B**).

### Node Degree of Subregions Within the Thalamus

**Figure 6:**
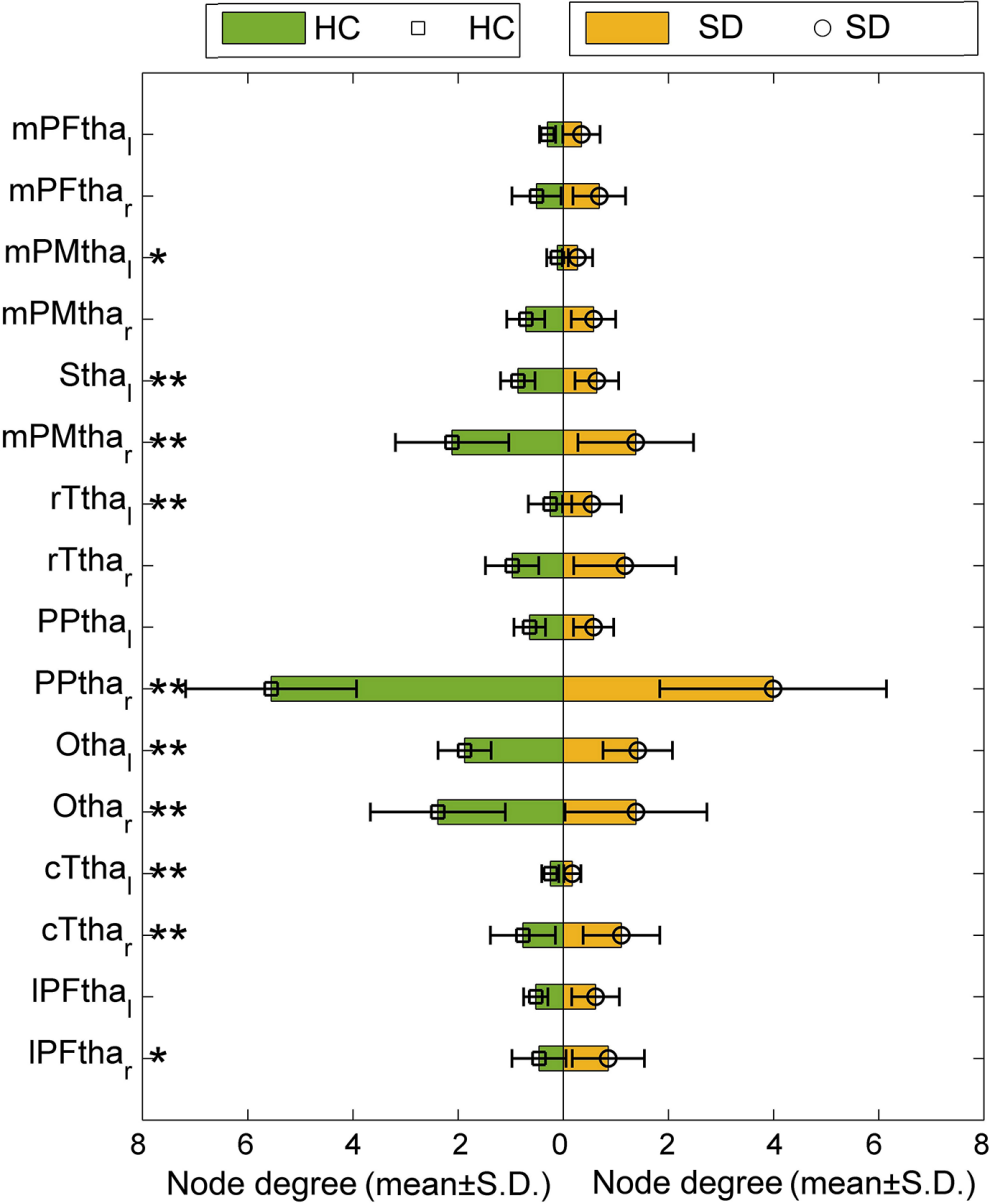
Node degree of 16 subregions in the thalamus of HC and SD.

The node degrees of 16 subregions within the thalamus are compared between SD and HC groups, as shown in **Figure 6**. Significant difference is found for 10 subregions. For eight subregions, the node degree of SD is significantly smaller than that of HC. The node degree of subregions on the right is higher than that of subregions on the left, for both SD and HC groups.

## Discussions

Sophisticated connectome-based brain biomarkers permit the association of brain measures with both subjective experiences and objective behaviors, leading to a reconceptualization of diagnoses of mental illness. Herein, we have built several reliable brain biomarkers (>0.9 accuracy) that predict SD using abnormal functional connections as input features and SVM as the machine learning algorithm. We have found dysfunctional brain regions, especially the thalamus and LHb, which may be the etiological origin of SD. We have observed a reduction of the node degree for the right LHb in SD, but not for the left. The significance of these findings and the related advantages of this methodology are interpreted and discussed in the following subsections.

### Reliable Biomarkers for Subclinical Depression Prediction

In this study, we have identified reliable brain biomarkers for SD prediction through the large-scale brain networks driven from resting state fMRI and a machine learning algorithm. Previously we had constructed biomarkers using the degree of centrality of different brain regions. The highest AUC was 0.82 for the right posterior parietal lobule (12). Here, the biomarkers are more reliable, and the highest AUC of 0.957 is achieved while using connections with the cingulate gyrus. Moreover, the arbitrary anatomically adjacent subregions (obtained by the sliding window method) and the anatomically well-defined subregions (obtained by the anatomical-region-based method) produce models with similar performances. These models present significantly higher accuracy than those driven by the randomly selected subregions. These results suggest that anatomical adjacency is important in the selection of feature (or connections) while building brain models or biomarkers. However, more sophisticated algorithms of feature selection, such as *L*_1_-regularized sparse canonical correlation analysis (*L*_1_-SCCA), may yield better biomarkers than anatomical adjacency (18, 25).

### Dysfunctional Brain Regions in Subclinical Depression

Using the criterion of owing prediction accuracy greater than 0.90, we identified the top five regions associated with dysfunction in SD from 24 cortical and subcortical regions, defined by the human Brainnetome Atlas (44). These regions include the thalamus, posterior superior temporal sulcus, cingulate gyrus, superior parietal lobule, and superior frontal gyrus. Most of these regions had been reported in previous studies of SD. The related findings for each dysfunctional region are described below.

Not unexpectedly, given that the thalamus has multiple functions of relaying information between different subcortical regions and the cerebral cortex, the dysfunctional thalamus is identified in SD. It had been reported that two subtypes of depression had hyperconnectivity between the thalamic and frontostriatal network, resulting in symptoms related to reward processing, adaptive motor control, and action initiation (27, 53). Of particular importance, LHb, a small epithalamic structure, is believed to control reward and aversion processing. The importance of these observations will be discussed in detail below.

Distinct connectivity patterns are observed in subregions of the posterior cingulate cortex for SD (8). The anterior cingulate cortex (ACC) is an important component of reward circuitry, with abnormalities resulting in anhedonia (loss of interest/pleasure), a core symptom in MDD (54). Abnormal ACC is also linked to default model network (DMN, self-related thoughts), hyperconnectivity, and switching between the DMN and the central executive network (CEN, externally-focused cognition) (8, 14, 55, 56).

Previously we had reported that the superior parietal lobule (SPL, Brodmann area 7, BA 7) presented the decreased fractional ALFF (fALFF) (9). The SPL had been proposed to be the key component controlling the executive network and playing a critical role in working memory (57).

The superior frontal gyrus includes the dorsolateral prefrontal cortex (DLPFC) and the medial prefrontal cortex (MPFC). In depression, DLPFC is used for emotion adjustment, with the activity of DLPFC inhibited at rest but increased during symptom remission (58, 59). Our previous work had shown that the functional connectivity between SPL and DLPFC was reduced in SD (9). MPFC is an important component of DMN playing a crucial role in self-referential processing. A lack of DMN inhibition, i.e., self-focus, is a core issue of MDD (60). Most importantly, both regions of the superior frontal gyrus had been the targets for repetitive transcranial magnetic stimulation (rTMS) in depression treatment (61).

### Dysfunctional Brain Regions Connected With the Thalamus

We have found that the dysfunctional thalamus in SD is mainly linked with the precuneus, insular gyrus, paracentral lobule, and amygdala. It is not surprising to observe the insula and amygdala because they are the neuroanatomical core of MDD pathology and closely related to anxiety (27). The precuneus is related to anhedonia, and the paracentral lobule (premotor) to anxiety. Positive connectivity between the LHb (an epithalamic structure) and the sensorimotor cortex had been reported by Ely et al. (13).

There is no overlap between the four regions with large number of abnormal connections associated with thalamus and the four regions (except thalamus) of the top five models ranked by accuracy. This is explained by the difference between machine learning and classical statistics which is discussed below.

### Lateral Habenula—Beyond a Reasonable Doubt

Only one previous study had investigated the resting state functional connectivity of the LHb in SD (13). Herein, the SD group shows greater LHb connectivity with DMN and lower connectivity with the salience network, which is consistent with prior finding in MDD. Here we found a lateralized decrease of node degree in the subregion with LHb (at right) in SD. This finding is consistent with our previous finding of decreased subcortical degree centrality (12). We speculate that the decreased node degree of LHb corresponds to its hyperactivity and abnormal bursts. Given that LHb has an inhibitory effect on dopamine neurons, Hikosak (32) proposed that the hyperactivity of LHb results in hypoactivity of dopamine neurons, reducing motor activity in MDD. Hyperactivity of LHb could be the result of bursts. According to Yang et al. (29), LHb burst firing increases in depression and LHb bursts lead to depression in rats. Interestingly, Ely et al. (13) found that LHb connectivity increased in the left and decreased in the right. This may partly explain the decreased node degree of the right LHb.

Among many dysfunctional brain regions, which one is the most likely etiological origin of SD? Is it LHb, as in depression for rats (29)? Given the correlative nature of resting-state fMRI, it is difficult to establish a causal inference (58). Therefore, we do not know which brain region is the cause or consequence of SD. However, based on these evidences given in our study, we believe that LHb may be the origin of SD, beyond a reasonable doubt.

### Machine Learning and Classical Statistics

In this study, we have used the classical statistical method, the two-sample *t*-test, to initially screen the candidate connections with significant difference between SD and HC groups. The selected connections are input into the SVM models as features. The two-sample *t*-test is actually used as a one feature selection algorithm. The method of using group tests has been proven to yield an inflated bias (62). Thus, we did not carry out strict multiple comparison corrections. More powerful feature selection or dimension reduction algorithms, such as L1-regularized sparse canonical correlation analysis, linear elastic-net, and minimum-redundancy maximum relevancy, can be assessed in the future (25, 63).

Moreover, we found that connections with small *p*-values do not always lead to high prediction accuracy in machine-learning-based models. This is consistent with previous studies, and originates from the essential difference between group difference and classification (62, 64, 65).

### Limitations and Future Works

The sample size is relatively small even though a large population is screened. The prediction accuracy may decrease with the sample size for most disorders (62). The generalizability of these models needs to be validated in independent cohorts. The 50 subjects (25 with high SD scores and 25 with low scores) from the WU-Minn Human Connectome Project (HCP) Consortium’s 500 Subjects Release (66), previously used by Ely et al. (13), will be used in the future.

Only the criterion of BDI-II score >13 is relatively simple compared with the complicated clinical evaluation of SD. However, there is no consensus among researchers regarding how to combine different scales to define SD. Besides the widely applied BDI, the National Institutes of Health (NIH) Toolbox Negative Affect Survey Sadness Subscale, and the Achenbach Adult Self-Report (ASR) have been utilized (13, 16). Regression model of BDI-II score may tackle the issue induced by the fixed threshold.

We only distinguished subjects with SD from HC. However, it is probable that SD has several neurophysiological subtypes, as shown in depression (27). With an increased sample size, it may be possible to differentiate these subtypes. Moreover, generalizing our identified biomarkers to distinguish between SD and MDD cannot be done before the specific verification. The study done by Lawson et al. (67) demonstrated that habenula function is also disrupted in MDD. In our group, the data of MDD is being collected so that the verification will be carried out in the near future.

The voxel size of our fMRI images is 3.5 × 3.1 × 3.1 mm, which is larger than the volume of LHb, approximately 18.5 mm^3^ per hemisphere (41). As a consequence the precise location of LHb is difficult to determine. The fMRI data with the whole brain coverage and high resolution (2 mm isotropic) is available for 25 subjects with high SD from the HCP (66). Using those high-resolution data, the node degree for LHb and its lateralization may be investigated clearly.

Actually, we have identified the dysfunctional brain regions from the predictive models driven by dysfunctional connections and especially identified LHb as the possible etiological origin of SD. To find the predictive and dysfunctional functional connections is also important. However, there are too many predictive functional connections in the current work to explain one by one. For example, even only for the right posterior parietal thalamus, 18 abnormal functional connections are available. Compared with the connections, the findings on brain regions are more reliable. Once the number of predictive functional connections is reduced to be less than 20 as done by Yahata et al. (25) their explanations will be feasible and valuable.

## Conclusion

Through integrating functional brain connections and SVM, this study has provided the connectome-based biomarkers for accurate prediction of SD. Abnormal brain connections with the thalamus and LHb are found to be implicated with SD. The right LHb in SD shows the decreased node degree comparing with HC group, but the left LHb does not. This evidence indicates that LHb may be the etiological origin of SD. The generated biomarkers can aid early diagnosis of SD. Furthermore, the identified dysfunctional brain connections and regions may help localize the etiological origin of SD and understand the pathogenesis of SD.

## Ethics Statement

This study was carried out in accordance with the recommendations of the guidelines of the Declaration of Helsinki, the Medical Ethics Committee of Guangzhou First People’s Hospital of Guangzhou Medical University. All subjects gave written informed consent in accordance with the Declaration of Helsinki. The protocol was approved by the Medical Ethics Committee of Guangzhou First People’s Hospital of Guangzhou Medical University.

## Author Contributions

SQ, JH, and XW designed and directed the study. YZ, SQ, BZ, DH, YT, and JH analyzed data. XW recruited participants and acquired data. YZ, SQ, and YT drafted the manuscript. All authors revised and approved the final version of the manuscript.

## Funding

The Fundamental Research Funds for the Central Universities (N181904003, N172008008), the National Science Foundation of China (81871846), and the Science and Technology Planning Project of Guangzhou (201804010032).

## Conflict of Interest Statement

The authors declare that the research was conducted in the absence of any commercial or financial relationships that could be construed as a potential conflict of interest.
